# Nipah Virus: A Zoonotic Threat Re-Emerging in the Wake of Global Public Health Challenges

**DOI:** 10.3390/microorganisms13010124

**Published:** 2025-01-09

**Authors:** Francesco Branda, Giancarlo Ceccarelli, Marta Giovanetti, Mattia Albanese, Erica Binetti, Massimo Ciccozzi, Fabio Scarpa

**Affiliations:** 1 Unit of Medical Statistics and Molecular Epidemiology, Università Campus Bio-Medico di Roma, 00128 Rome, Italy; 2 Department of Public Health and Infectious Diseases, University of Rome Sapienza, 00161 Rome, Italy; giancarlo.ceccarelli@uniroma1.it (G.C.); dott.albanese.mattia@gmail.com (M.A.); erica.binetti@uniroma1.it (E.B.); 3 Internal Medicine, Endocrine-Metabolic Sciences and Infectious Diseases, Azienda Ospedaliero Universitaria Umberto I, 00161 Rome, Italy; 4 Migrant and Global Health Research Organization—Mi-Hero, Italy; 5 Department of Science and Technologies for Sustainable Development and One Health, Università Campus Bio-Medico di Roma, 00128 Rome, Italy; giovanetti.marta@gmail.com; 6 Instituto René Rachou, Fundação Oswaldo Cruz, Belo Horizonte 30190-002, Brazil; 7 Climate Amplified Diseases and Epidemics (CLIMADE)—CLIMADE Americas, Belo Horizonte 30190-002, Brazil; 8 Hospital of Tropical Diseases, Mahidol University, Bangkok 10400, Thailand; 9 Department of Biomedical Sciences, University of Sassari, 07100 Sassari, Italy; fscarpa@uniss.it

**Keywords:** Nipah virus, zoonotic pathogens, global health, surveillance

## Abstract

The re-emergence of the Nipah virus (NiV) in Kerala, India, following the tragic death of a 14-year-old boy, underscores the persistent threat posed by zoonotic pathogens and highlights the growing global public health challenge. With no vaccine or curative treatment available, and fatality rates as high as 94% in past outbreaks, the Nipah virus is a critical concern for health authorities worldwide. Transmitted primarily through contact with fruit bats or consumption of contaminated food, as well as direct human-to-human transmission, NiV remains a highly lethal and unpredictable pathogen. The World Health Organization has classified Nipah as a priority pathogen due to its alarming potential to cause widespread outbreaks and even trigger the next pandemic. Recent outbreaks in India and Bangladesh, occurring with seasonal regularity, have once again exposed the vulnerability of public health systems in containing this virus. This study explores the epidemiology, ecological factors driving transmission, and the public health response to NiV, emphasizing the role of zoonotic spillovers in pandemic preparedness. As the global community grapples with an increasing number of emerging infectious diseases, the Nipah virus stands as a stark reminder of the importance of coordinated surveillance, rapid containment measures, and the urgent development of novel strategies to mitigate the impact of this re-emerging threat.

## 1. Introduction

Nipah virus (NiV) is a highly pathogenic, zoonotic virus belonging to the family *Paramyxoviridae* and the genus *Henipavirus* [1]. First identified during an outbreak in Malaysia and Singapore in 1998–1999, NiV has since been associated with sporadic yet severe outbreaks in South and Southeast Asia, particularly in Bangladesh and India [2]. The Nipah virus genome consists of single-stranded, negative-sense RNA, approximately 18.2 kb in length, encoding six structural proteins: nucleocapsid (N), phosphoprotein (P), matrix (M), fusion (F), glycoprotein (G), and large polymerase (L) [2]. These proteins are essential for viral replication, host immune evasion, and the transmission process, allowing the virus to infect a broad range of mammalian hosts, with fruit bats (*Pteropus* species) identified as the natural reservoir [2].

Epidemiologically, NiV outbreaks are seasonal in nature, with a high prevalence in regions where close contact between humans and fruit bats occurs, often through the consumption of date palm sap contaminated with bat excreta [3]. Human-to-human transmission is also well documented, notably in healthcare settings, further elevating the public health risks. Infected individuals typically present with a range of symptoms, from fever and respiratory distress to fatal encephalitis, with case fatality rates fluctuating between 40% and 94%.

Phylogenetic studies of NiV have revealed two genetically distinct lineages—Malaysian and Bangladeshi—with the latter exhibiting greater genetic heterogeneity [3]. Notably, Bangladeshi NiV genomes were found to be six nucleotides longer than their Malaysian counterparts, highlighting ongoing virus evolution. These findings underscore the importance of genomic surveillance in understanding viral diversity and tracking transmission pathways during outbreaks [3]. The ecological dynamics of Nipah virus are closely linked to the habitat and behavior of fruit bats, particularly *Pteropus vampyrus* in Southeast Asia and *Pteropus medius* in South Asia [4]. These bats, which roost in a variety of environments, have been driven closer to human populations due to urbanization, deforestation, and industrial activities, increasing the risk of zoonotic spillover [5].

The World Health Organization (WHO) classified NiV as a priority pathogen in 2018 due to its high lethality, zoonotic transmission potential, and pandemic threat, stressing the need for intensified global surveillance efforts. The unpredictability of NiV outbreaks, coupled with the lack of specific antiviral treatments or vaccines, poses a significant global health challenge, as recognized by the World Health Organization (WHO), which has listed NiV among its priority pathogens due to its potential to cause widespread pandemics [6]. The ongoing threat of zoonotic spillover events, exemplified by the recent re-emergence of NiV in Kerala, India, underscores the critical need for robust surveillance systems [7]. Zoonotic infections like Nipah require integrated approaches combining ecological, epidemiological, and genomic data to better understand transmission dynamics and mitigate risks.

Genomic surveillance, in particular, offers invaluable insights into the virus’s evolution, transmission routes, and the emergence of new strains, enabling more effective outbreak responses. The aim of this study is to integrate genomic and epidemiological data to provide a comprehensive analysis of the recent Nipah virus re-emergence in India. By combining real-time genomic sequencing with detailed epidemiological investigations, this study seeks to elucidate transmission patterns, identify potential spillover events, and assess the effectiveness of current surveillance and containment strategies. Understanding these factors is crucial for developing targeted interventions and improving global preparedness for future outbreaks.

## 2. Geographical Distribution of NiV Outbreaks and Epidemiological Insights

The emergence of NiV as a significant public health threat can be traced back to its first documented outbreak in Peninsular Malaysia between September 1998 and May 1999. This first outbreak, which resulted in 265 cases of acute encephalitis and 105 deaths, marked the introduction of the virus to the scientific community and highlighted its devastating potential [8]. The outbreak mainly affected pig farmers and people in close contact with infected pigs, leading to the slaughter of over one million pigs to contain the spread. Concomitant cases in Singapore among slaughterhouse workers handling pigs imported from Malaysia further demonstrated the virus’s ability to spread geographically through the cattle trade [9]. 

The pattern of NiV outbreaks has changed significantly since its initial identification, with Bangladesh and India becoming major hotspots for recurrent outbreaks. Since 2001, Bangladesh has experienced almost annual outbreaks, with a distinct epidemiological pattern characterized by sporadic cases and small clusters, mainly linked to the consumption of raw date palm sap contaminated by infected bats [10]. These outbreaks follow a clear seasonal pattern, typically occurring between December and April, coinciding with the date palm sap collection period. During these months, *Pteropus* bats, the primary source of zoonotic transmission, are more active near human settlements, thereby increasing the risk of contamination and infection [11]. The Indian state is another significant area for NiV transmission, the first documented outbreak of which occurred in Siliguri, West Bengal, in 2001, with 66 cases and a 74% mortality rate. This outbreak was particularly noteworthy for demonstrating efficient human-to-human transmission in a hospital setting [12]. The state of Kerala, India, has emerged as a major hotspot for NiV outbreaks, with multiple incidents reported since 2018. The initial outbreak in May 2018 was particularly severe, resulting in 17 deaths out of 18 confirmed cases, with a staggering 94.4% mortality rate. Subsequent outbreaks in 2019, 2021, and 2023 reinforced the endemic potential of NiV in the region [13]. The latest outbreak, in September 2023, occurred in Kozhikode district following the death of a 14-year-old boy, again underscoring the persistent threat posed by the virus and the challenges to control its spread. Recent years have seen a broadening of the understanding of the potential geographical range of NiV, with evidence of viral circulation in fruit bats in wider regions of South and Southeast Asia than previously recognized. Serological studies have detected antibodies to NiV in bat populations in Thailand, Cambodia, Indonesia, and the Philippines, suggesting a wider distribution of the virus and the possibility of future outbreaks in new areas [14].

**Figure 1:**
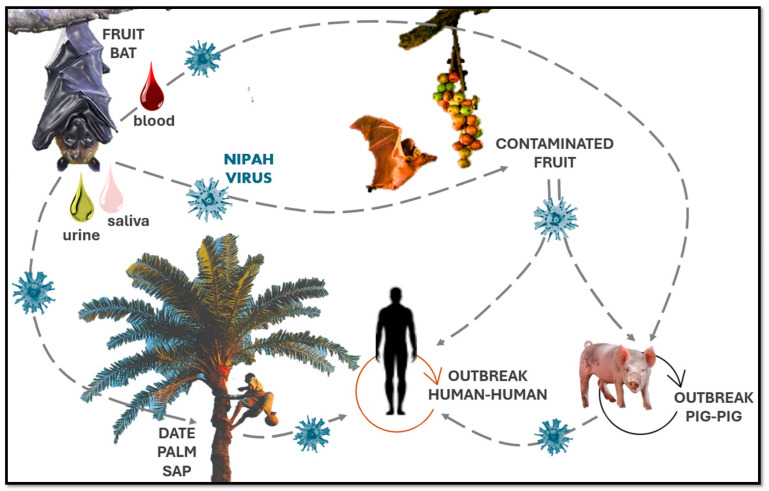
NiV transmission cycle.

Effective outbreak control requires a multifaceted approach, including rapid case identification, contact tracing, isolation measures, and, when possible, pre-emptive vaccination of at-risk groups [15]. However, the unpredictable nature of spillover events and the potential for human-to-human transmission continue to pose significant challenges for outbreak control and prevention. Transmission of NiV is characterized by zoonotic spillover from animal hosts, particularly fruit bats of the genus *Pteropus*, and human-to-human spread, which can occur in healthcare settings and between close contacts, as shown in Figure 1. The virus circulates asymptomatically in *Pteropus* bats, which spread NiV through saliva, urine, and feces. NiV can then infect intermediate hosts, such as pigs, or pass directly to humans through food or environmental contamination.

For example, zoonotic transmission in Bangladesh is often due to the consumption of raw date palm sap contaminated by bats. This local food practice, in which date palm sap is tapped from trees into open containers, presents a high risk of contamination, as bats are known to feed on this sap and may leave saliva or other fluids on the collection vessels [16]. In Malaysia, pig farming was the main amplifying host of human cases, resulting in the rapid spread of the virus among pig farmers. Since then, the spread of the virus has been linked to different animals and human activities depending on local conditions, and the experience in Malaysia has prompted changes in livestock management practices to prevent similar incidents [17]. Human-to-human transmission has emerged as a critical route of Nipah virus spread in Bangladesh and India, particularly within healthcare settings, where hospital clusters have been documented. The 2001 outbreak in Siliguri, India, highlighted this risk, with the virus spreading extensively among patients, healthcare workers, and family members [12]. Similar clusters were observed during the 2018 and 2023 Kerala outbreaks, where direct contact with infected body fluids facilitated transmission. 

Environmental and anthropogenically driven changes have also played a crucial role in shaping NiV epidemiology. Deforestation, agricultural expansion, and urbanization reduce natural habitats for *Pteropus* bats, bringing them into closer proximity to human populations. These bats adapt to human-altered environments, where they may seek food in agricultural lands or near residential areas, thus increasing the likelihood of zoonotic spillovers. For example, in Bangladesh, deforestation has decreased available habitats for bats, leading to higher interactions with people in rural settings, especially where date palm sap collection is common [18,19]. Climate change may also influence NiV trans-migratory patterns and seasonal behaviors. For example, changes in temperature and precipitation can affect fruit and flowering seasons, altering bat feeding habits and potentially changing the timing and location of human–bat interactions. While the exact effects of climate change on NiV transmission remain under study, it is likely that climate-related shifts in bat behavior could impact the virus’s spread across different regions and seasons [20,21].

Molecular studies have provided insights into NiV’s evolution and pathogenic potential. Two primary genetic strains of NiV have been identified: the Malaysian strain, which was responsible for the initial outbreak [22], and the Bangladeshi strain, associated with more recent cases in Bangladesh and India. The Bangladeshi strain has demonstrated greater genetic variability and higher virulence, with mortality rates exceeding 75% in some outbreaks [23]. This strain’s ability to spread through human-to-human transmission in healthcare settings underscores its elevated public health risk compared to the Malaysian strain, which has shown limited human-to-human transmission [23]. These molecular distinctions have significant implications for NiV surveillance and diagnostic strategies. Genomic monitoring helps public health authorities track the virus’s evolution and potential changes in transmission dynamics, which may inform vaccine and therapeutic development [24]. For instance, the greater genetic diversity observed in the Bangladeshi strain suggests a need for diagnostics that can accurately detect both strains to enable effective outbreak response and management [24]. Managing NiV requires continued surveillance, especially in regions with significant bat populations and established human cases [25,26]. Research efforts are underway to develop monoclonal antibodies and antiviral drugs that can target NiV [27,28]. Additionally, experimental vaccines are being tested to confer immunity in high-risk populations, though further clinical trials are necessary to ensure efficacy and safety [29,30]. Integrating ecological and genetic data into epidemiological models may improve predictive capabilities for future outbreaks, enabling public health systems to allocate resources effectively and prevent spillover events [23,31]. In areas where the Nipah virus has spread, such as Bangladesh and Kerala, ongoing public education and culturally sensitive interventions are key to reducing human exposure and mitigating the impact of the virus. In Bangladesh, for example, culturally appropriate interventions have been developed to prevent person-to-person transmission of NiV, emphasizing the importance of community involvement and understanding of local practices [32]. Similarly, during the NiV outbreak in Kerala (https://gvn.org/update-on-the-nipah-virus-outbreak-in-kerala-india/?utm_source=chatgpt.com, accessed on 28 December 2024), India, experts emphasized the need for culturally sensitive educational campaigns aimed at the affected community to effectively contain the virus.

The risk of NiV spread in global regions with *Pteropus* bat populations, such as Australia and Africa, is an area of growing scientific interest. In Australia, *Pteropus* bats, known as flying foxes, occur in several regions. A study qualitatively assessed the risk of introduction and establishment of NiV in Australian populations of flying foxes through movements of these animals from neighboring regions, such as eastern Indonesia, East Timor, and Papua New Guinea. The results indicate that the probability of NiV establishment through migratory or non-migratory routes is “very low”, albeit with a high degree of uncertainty [33]. In addition, rapid changes in bat ecology, driven by factors such as changes in land use, could influence the dynamics of bat-borne viruses, as evidenced by studies of Hendra virus in Australia [34]. These dynamics could have implications for the transmission of other zoonotic viruses, including NiV. In Africa, although no cases of NiV infection in humans have been documented, the presence of *Pteropus* and other frugivorous bats raises concerns about the potential risk of spread. Antibodies against Nipah and Hendra viruses have been detected in frugivorous bats of the genus Eidolon in Africa, suggesting that these viruses, or related viruses, may be present in the geographic range of *Pteropus* bats in Africa. A critical review [35] analyzed the current knowledge of African bats as reservoirs of viruses, including paramyxoviruses such as NiV, highlighting the need for further research to better understand the associated risks.

## 3. Phylogenetic Analysis

**Figure 2:**
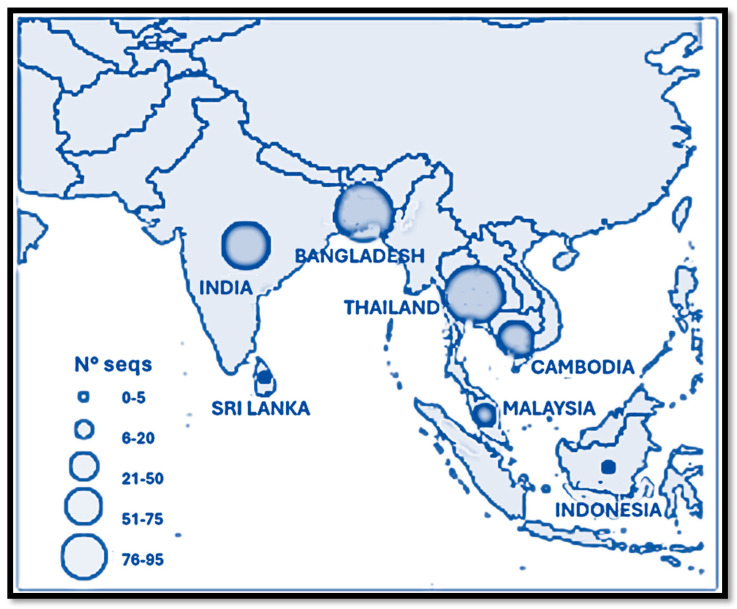
Geographic distribution of NiV sequences by country. This map illustrates the geographic distribution of NiV sequences, represented by circles proportional to the number of sequences collected from each country. The legend indicates the range of sequence counts for each circle size, from 0–5 to 76–125. Countries included in the dataset are Bangladesh, Cambodia, India, Indonesia, Malaysia, Sri Lanka, and Thailand. These sequences reflect the temporal and host-specific sampling efforts, emphasizing the regions most affected by NiV outbreaks and the importance of genomic surveillance to better understand transmission dynamics and risks.

In order to perform an upgrade on the evolutionary pathway of all lineages, a phylogenetic reconstruction was performed on a dataset including all available whole genomes of *Henipavirus nipahense* from NCBI Virus (https://www.ncbi.nlm.nih.gov/labs/virus/vssi/#/, accessed on 28 December 2024). The selection of included genomes was carried out without applying arbitrary criteria, in order to avoid potential biases in sampling. The only filter applied concerned genome quality and genomic coverage relative to the reference sequence (NC_002728.1). Specifically, only high-quality genomes were included, while those with genomic coverage below 75% were excluded to prevent the introduction of an excessive number of non-informative sites that could negatively impact the analysis. After alignment and manual editing, performed using the software MAFFT v.7.505 [36] and UGenePro v.35 [37], respectively, the dataset of 95 whole genomes was processed for the reconstruction of the phylogenetic relationships using the software MrBayes v. 3.2.7 [38]. This dataset includes sequences from Bangladesh, Cambodia, India, Indonesia, Malaysia, Sri Lanka, and Thailand, as shown in Figure 2, highlighting the geographic diversity of sampling efforts critical for understanding the phylogenetic relationships and evolutionary dynamics of the virus.

Two independent runs were conducted, each employing four Metropolis-coupled Markov-chain Monte Carlo (MCMCMC) simulations, consisting of one cold chain and three heated chains. Both analyses ran concurrently for 5,000,000 generations, with trees being sampled every 1000 generations. To account for burn-in, the first 25% of the total 10,000 sampled trees were excluded. Nodes with posterior probabilities exceeding 0.95 were regarded as statistically robust. The final phylogenetic tree was visualized using FigTree version 1.4.0 [39].

**Figure 3:**
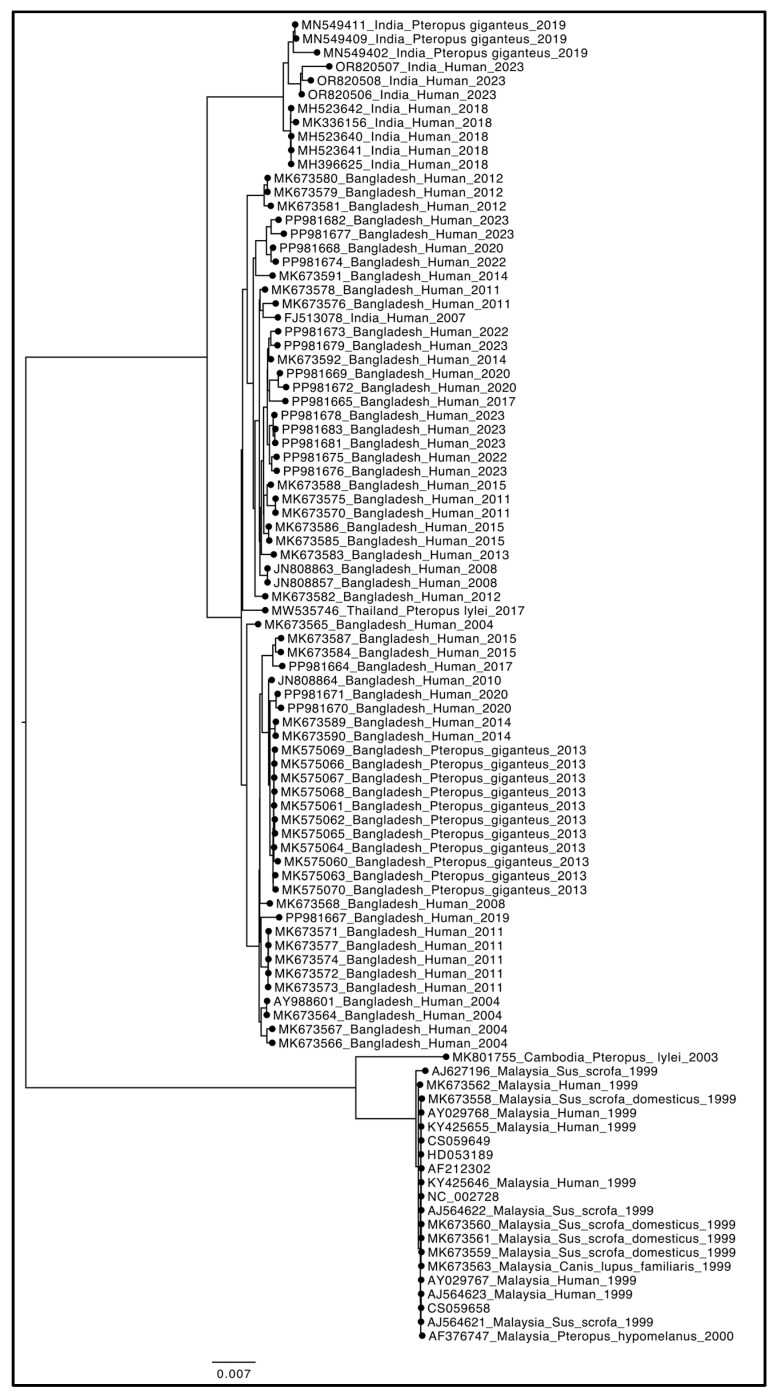
Phylogenetic reconstruction of *Henipavirus nipahense*. Bayesian phylogenetic tree of *n* = 95 genome sequences available on NCBIVirus as of 30 November 2024. All nodes are fully supported for posterior probabilities. The branch lengths of the clades were cropped to fit the page while maintaining proportions. The image was edited using GIMP 2.8 (available at https://www.gimp.org/downloads/oldstable/, accessed on 2 December 2024).

The phylogenetic tree shown in Figure 3 revealed a strong genetic structure, dividing the entire dataset into two main clades, both fully supported statistically. The first clade includes the oldest samples (such as the reference genome NC_002728), collected in 1999, along with two additional records from 2000 and 2003. The second clade, more represented in terms of sample size and genome prevalence, consists of more recent isolates spanning a broader collection range, from 2004 to 2023.

**Figure 4:**
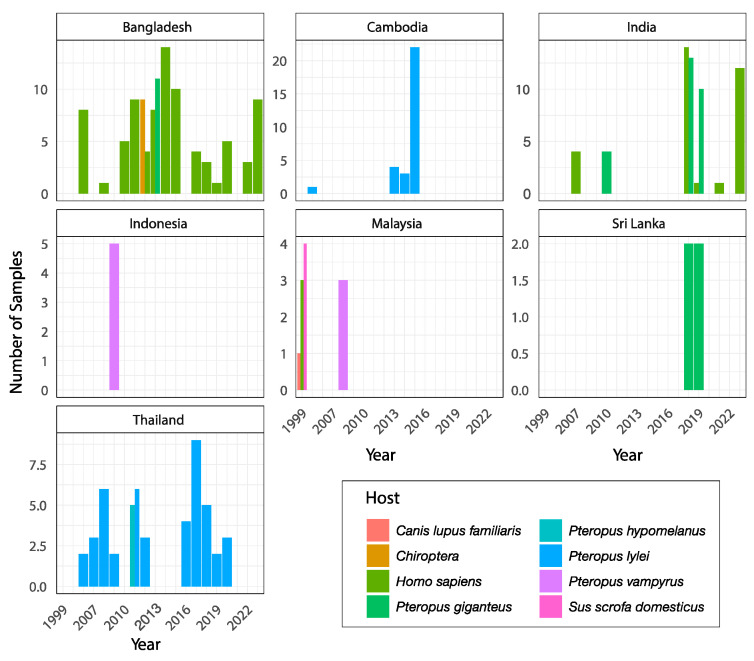
Geographic and host distribution of NiV samples over time. This figure presents the temporal distribution of Nipah virus isolates across different countries (Bangladesh, Cambodia, India, Indonesia, Malaysia, Sri Lanka, and Thailand) and their associated host species. The *x*-axis represents the year of sampling, while the *y*-axis indicates the number of samples. Bars are color-coded to represent the host species, including *Pteropus* bats (e.g., *P. vampyrus*, *P. giganteus*, *P. hypomelanus*, and *P. lylei*), humans (*Homo sapiens*), and other species such as dogs (*Canis lupus familiaris*) and pigs (*Sus scrofa domesticus*). In cases where bats were not identified to the species level, the bar is labeled with Chiroptera, indicating the taxonomic order for bats. This distribution highlights the diversity of host species and geographic regions associated with Nipah virus spillover events and outbreaks, emphasizing the need for targeted genomic and ecological surveillance efforts to mitigate future risks.

It is interesting to note that there is no consistent host-specific structuring, as genomes isolated from humans clustered together in both clades, indicating a lack of genetic differentiation that could have driven host adaptation. The Nipah virus is primarily transmitted by fruit bats (*Pteropus* spp.), making the zoonotic context essential. The absence of host-specific structuring observed in the phylogeny suggests that spillover events from natural hosts (bats) to humans are likely independent and repeated occurrences. This emphasizes the need for ecological investigations into reservoirs and the factors that facilitate these transmissions. The lack of specific adaptation to human hosts in the genomes of isolated NiV strains indicates that human infection is not the result of co-evolution but rather of accidental and recent spillover events. This aligns with the current knowledge of Nipah, where outbreaks are zoonotic in origin and human-to-human transmission is limited compared to the initial zoonotic spillover. The two clades observed in the phylogenetic analysis might reflect geographic or ecological differences among bat populations, as seen in NiV strains from Bangladesh and Malaysia [40]. As illustrated in Figure 4, the distribution of Nipah virus isolates across different host species and countries provides further insight into the diversity of ecological contexts where spillover events occur. This figure underscores the significance of integrating genomic and ecological data to identify key reservoirs or spillover pathways, as well as to inform targeted surveillance efforts.

This could have implications for understanding the geographic spread of the virus and the variations in lethality or transmissibility across regions. The genetic structure and distribution of isolates across clades have important implications for public health strategies. Understanding the ecological or geographic origins of the basal clade could help identify key reservoirs or spillover pathways, aiding in targeted surveillance efforts. The genetic differentiation observed between clades might reflect regions or periods where the virus evolved under distinct selective pressures, providing critical insights for predicting future outbreaks and assessing the risk of human adaptation. Additionally, the apparent lack of host adaptation in both clades highlights the importance of monitoring potential shifts in genetic structure that could indicate the emergence of human-adapted variants with pandemic potential. The older clade appears to carry ancient genetic variability, as evidenced by its long branch length and substantial distance in terms of the number of mutations. Its basal position in the phylogenetic tree suggests that this clade may represent the origin of the current genetic variability. The basal clade, with older samples, may represent the original strain responsible for early outbreaks in Malaysia, while the more recent clade could be associated with strains isolated in India and Bangladesh.

The presence of older samples, both human and animal, in the basal clade suggests that it represents an evolutionary archive of the Nipah virus from the early outbreaks. Not only is this long branch length the result of prolonged evolution in the natural reservoir, but also it reflects the ancestral position of these strains in relation to the later diversification seen in more recent clades. The coexistence of human and animal samples within this basal clade further implies that the first zoonotic transmissions likely occurred without the virus acquiring specific adaptations to human hosts. This supports the hypothesis that human infections were not the result of co-evolution but rather of sporadic spillover events, driven by ecological or behavioral factors. Moreover, the persistence of this basal clade, with genetically distinct characteristics compared to more recent clades, suggests that the strains involved in early outbreaks in Malaysia and elsewhere did not become extinct but remained confined in animal populations or ecologically isolated regions. Another consideration is the temporal representation of these strains. The fact that the basal clade only includes older samples could indicate a decline or replacement of these strains over time, possibly due to the emergence of more recent variants within the subsequent clade. This shift might have been driven by natural selection, ecological pressures, or changes in the dynamics of the natural reservoir. The basal clade might, therefore, represent a lineage that was once more prevalent but has since been overshadowed by newer, more virulent strains.

Furthermore, the inclusion of animal samples in the basal clade reinforces the need to explore the relationship between current strains and these older variants. Understanding whether the virus has remained genetically stable within the natural reservoir or whether the basal clade represents a lineage that did not undergo significant geographical expansion or adaptation is crucial. Such an analysis is important to assess whether strains similar to those in the basal clade could still pose a latent zoonotic threat, and under what conditions they might re-emerge. These findings highlight the need for continuous surveillance of the virus in both human and animal populations to monitor for potential re-emergence of older strains or the evolution of new variants with increased human-to-human transmission potential.

## 4. Clinical Presentation

### 4.1. Clinical Features

Studies report varying incubation periods for NiV, ranging from 4 to 21 days [1]. Some studies suggest a longer timeframe, up to two months, including late-onset encephalitis cases [41]. In 92% of cases, symptoms appear within the first two weeks, with an average onset of 10 days [42].

Table 1 provides a comprehensive overview of the key clinical differences between NiV-M and NiV-B, aiding in understanding the distinct characteristics of each strain and their implications for patient management.

**Figure 5:**
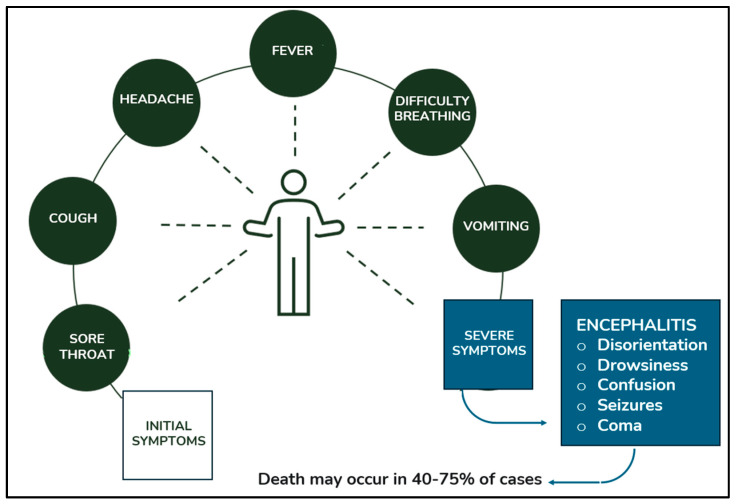
Nipah virus infection can manifest in a range of symptoms, from mild illness to severe encephalitis, and can be fatal.

Early symptoms are often nonspecific (fever, vomiting, headache, dizziness), making differentiation from other illnesses challenging. As the disease progresses, clinical features may worsen, involving neurological and/or respiratory systems. In some cases, other organs (myocardium, pancreas, kidneys) may also be affected (Figure 5) [46]. Following an initial phase of flu-like symptoms (fever, cough, myalgia), the disease can progress to atypical pneumonia [2], characterized by breathlessness, tachypnea, and chest X-ray infiltrates [42]. Severe cases may develop into ARDS, requiring mechanical ventilation. Nipah–Bangladesh infections appear more likely to present with respiratory symptoms than Nipah–Malaysia infections [43]. Neurological signs and symptoms are common across all NiV variants. Encephalitis may develop acutely or subacutely, with potential late onset or relapses years later. Therefore, physicians should not solely focus on recent travel history when gathering patient information [47]. Common neurological symptoms include reduced consciousness, areflexia, brainstem dysfunction (tachycardia, hypertension, abnormal pupils, doll’s eye reflex), behavioral changes, and seizures. Patients with late-onset or relapsing encephalitis may initially present with asymptomatic NiV infection or absence of encephalitis, possibly due to less severe infection. However, minimal brain lesions characteristic of NiV might be visible on MRI. Clinical manifestations resemble acute onset, with prominent seizures and focal cortical signs [48]. Extensive vasculitis has been observed in various organs, most commonly affecting small arteries and arterioles in the CNS [49]. This can result in long-term neurological and functional morbidity. Late-onset or relapsing cases experience more frequent sequelae than acute encephalitis cases (61% vs. 22%), but with lower mortality (18% vs. 40%) [50].

### 4.2. Diagnosis

Diagnosis of NiV infection can be challenging due to the nonspecific nature of early symptoms. Additionally, serological evidence may only be detectable later, as IgM antibodies typically develop approximately 15 days after infection onset [51]. Consequently, to control outbreaks, the Indian National Centre for Disease Control has developed guidelines to enhance case identification. A “suspected case” is defined as any individual presenting with neurological or respiratory symptoms—such as headache, altered mental status, seizures, cough, or shortness of breath—from a community experiencing an outbreak. These guidelines also define “close contacts” to facilitate identification of individuals requiring 21 days of follow-up to prevent further spread [52]. In Bangladesh, India, Malaysia, and Singapore, particular attention should be given to individuals with compatible symptoms who have had contact with bats, sick animals, livestock, raw date palm juice, or sap [53].

Various epidemiological studies have developed mathematical models to predict NiV outbreaks. Classical mathematical models and basic fractional/fractal fractional theory have been employed to understand the disease’s epidemiology and predict/manage potential outbreaks [54,55].

Regarding viral isolation, NiV grows in Vero cells, exhibiting a paramyxovirus-like cytopathic effect within three days (though five days are required to rule out growth). Viral isolation can be performed on various human specimens, including nasal/throat swabs, urine, blood, and CSF. NiV has also been isolated from the kidneys, lungs, and spleen of infected animals [56].

The virus can be identified through electron microscopy or immunoelectron microscopy. Common identification methods include immunostaining/fixation of infected cells, immunohistochemistry, RT-PCR, VNT (virus neutralization test), and qRT-PCR [57]. Recent studies highlight the potential for early diagnosis by detecting the viral N gene using a one-step RT-ddPCR assay, even at low viral loads [58]. Another promising approach involves detecting NiV RNA in saliva during the acute phase, offering a non-invasive and easily collected specimen [1].

Regarding serological investigations, after the discovery of NiV’s similarity to Hendra virus, it was found that HeV antibody tests could also detect NiV antibodies. For example, an ELISA designed for HeV was adapted in Malaysia for pig farm surveillance by substituting NiV antigens for HeV antigens [56]. Studies on seroconversion kinetics in NiV infections indicate that IgM antibodies are detectable in 44–50% of cases on day 1, reaching 100% positivity by day 12, and persisting for up to three months post-infection. IgG antibodies appear later, with low positivity until day 10, reaching 100% by days 25–26, and persisting for at least eight months [59]. Therefore, while RT-PCR is the preferred method for early diagnosis, antibody testing is suitable for later stages, epidemiological studies, and seroprevalence surveys.

Antibody detection is performed using ELISA on blood and CSF. Early tests employed gamma-irradiated NiV antigens, but various tests have since been developed [5]. Specifically, ELISA using recombinant NiV-N protein has demonstrated good sensitivity and specificity, comparable to the CDC’s standardized test [60]. High specificity has also been observed with antigen-capture ELISA using the anti-N antibody 1a11c1 [61]. Microsphere suspension array assays (solid-phase blocking ELISA) offer another diagnostic option. Despite lower sensitivity and specificity compared to other ELISAs, their lower cost and multiplexing capabilities make them potentially valuable for resource-limited settings. However, this test should be considered a screening tool requiring confirmation with a serum neutralization assay [62].

Regarding biosafety, NiV is classified as a Risk Group 4 pathogen, requiring handling in BSL-4 laboratories for procedures like virus propagation, isolation, quantification, and neutralization. To expedite initial laboratory protocols during suspected outbreaks, diagnostic procedures can be performed in Physical Containment Level 3 facilities until etiological confirmation. Serological tests and RT-PCR can be conducted at BSL-2 if samples are inactivated [63].

### 4.3. Treatment and Management

Currently, no approved treatment exists for Nipah virus infection. Clinical management primarily consists of supportive care, encompassing oxygen and fluid administration, nutritional support, anticonvulsant and antipyretic medications, and empiric therapy. This care is typically provided in an intensive care unit, particularly for patients experiencing severe respiratory or neurological complications. Various therapeutic strategies have been proposed thus far, with mixed results [44].

While direct antiviral treatments are lacking, some existing medications have been explored for their potential to combat NiV. Ribavirin, an antiviral drug, has shown some in vitro activity against the virus, but its effectiveness in treating human infections remains [64]. Research is ongoing to identify and develop more effective antiviral therapies. Monoclonal antibodies, specifically designed to target the Nipah virus, are also under investigation as a potential treatment [64]. These antibodies could help neutralize the virus and prevent further spread [65].

The development of effective treatments for NiV infection is a critical area of research. Given the virus’s high mortality rate and potential for outbreaks, finding ways to directly target and eliminate the virus is essential. This research involves exploring various antiviral strategies, including the development of new drugs and the repurposing of existing ones. The ultimate goal is to have safe and effective treatments readily available to combat NiV infection and improve patient outcomes. Therapeutic strategies explored to date are reported in Table 2.

The therapeutic window for remdesivir and other antivirals is narrow, suggesting their potential use as prophylaxis or early treatment. On the other hand, m102.4, targeting the Hendra virus G glycoprotein, is the only one tested in humans (Phase 1 trial). It has been used as post-exposure prophylaxis for Hendra virus in Australia (14 patients) and NiV in India, with febrile reaction as the only observed adverse effect [74].

### 4.4. Prevention

Nipah virus transmission occurs through several routes, with considerable attention focused on the consumption of raw date palm sap in regions where this practice is common. Bats, the natural reservoir of NiV, contaminate collection pots by licking the sap-producing surfaces of the date palms. This is considered the primary infection route in affected areas, leading to a seasonal pattern of outbreaks typically between January and May, coinciding with the sap collection period [12]. Informational campaigns promoting the use of bamboo skirts on date palms to prevent bat contact have been implemented to mitigate this risk [84]. Person-to-person transmission is another significant concern. Prevention measures include isolating infected or potentially infected individuals for 21 days and implementing robust surveillance and contact tracing systems. Healthcare workers should adhere to standard infection control protocols, including glove use, hand hygiene, and appropriate personal protective equipment. Pigs serve as intermediate hosts, and close contact with infected animals has been identified as a transmission route in past outbreaks. Pigs can contract the virus by consuming bat saliva-contaminated fruits. Other domestic animals, including sheep, goats, dogs, cats, and horses, have also tested positive for NiV. Contact with these animals, particularly during slaughter, poses a high transmission risk to humans. Precautionary measures such as wearing gloves and appropriate protective clothing are crucial [1].

### 4.5. Vaccination

While no licensed vaccines are currently available for human use against Nipah and Hendra viruses, significant research efforts are underway, with several vaccine candidates in clinical trials [65]. One vaccine is currently approved for horses [85]. Various strategies are being explored for NiV and HeV vaccine development [86]. Several viral vectors have been investigated, including poxviruses, adenoviruses, rhabdoviruses, paramyxoviruses, and Venezuelan equine encephalitis virus [87]. Recombinant vesicular stomatitis virus vectors have shown promising results, inducing high titers of HeV G-specific antibodies, surpassing those elicited by recombinant rabies virus vectors. This approach leverages the safety and established track record of rhabdoviral vaccine vectors [88]. A subunit vaccine based on the HeV glycoprotein has demonstrated high immunization levels against both HeV and NiV in various animal models (cats, ferrets, African green monkeys). This is currently the only licensed HeV vaccine approved for veterinary use in Australia [85].

Virus-like particles represent another promising strategy. NiV-VLPs, composed of the M, G, and F proteins, have conferred high levels of protection in Golden Syrian hamsters [89]. Peptide-based NiV vaccines, utilizing epitopes mimicking the N, V, and F proteins, have been developed to stimulate T-cell responses [90]. Epitopes for the G and M proteins, capable of binding both B-cells and T-cells, have also been synthesized [91]. More recently, mRNA vaccines have emerged as a potential platform due to their safety and ease of production. Table 3 highlights the key aspects of mRNA-based vaccines against Nipah virus, emphasizing their potential, challenges, and the ongoing efforts to bring them to clinical and regulatory approval. The rapid adaptability of mRNA platforms holds promise for addressing future NiV outbreaks efficiently. Currently, while an HeV-sG mRNA vaccine showed limited efficacy, another mRNA vaccine candidate, mRNA-1215, is under development, employing a similar approach to COVID-19 mRNA vaccines [65].

## 5. Discussion and Conclusions

The recent re-emergence of the NiV in Kerala, India, has once again brought the world’s attention to the persistent challenge posed by zoonotic pathogens. This incident not only underscores the high lethality of NiV but also highlights systemic vulnerabilities in global public health systems [92]. Here, we delve into the implications of these outbreaks and explore avenues for improving pandemic preparedness and response. The transmission of NiV underscores the critical role of zoonotic spillovers in shaping the epidemiology of emerging infectious diseases. The natural reservoir of the virus, *Pteropus* fruit bats, serves as a continuous source of infection, with spillovers facilitated by human activities such as deforestation, agricultural expansion, and urbanization. These factors exacerbate human–wildlife contact, increasing the risk of transmission to humans either directly or via intermediate hosts such as livestock. Additionally, the seasonal nature of outbreaks observed in regions like India and Bangladesh highlights the influence of ecological cycles, such as the availability of fruiting trees or specific climatic conditions, which warrant further investigation to refine predictive models for outbreak risk. The high case fatality rate (CFR) of NiV, which can reach up to 94%, combined with its capacity for human-to-human transmission, presents a daunting challenge for public health systems, particularly in resource-limited settings [93]. The outbreak in Kerala exemplifies the dual burden faced by health authorities: simultaneously managing the immediate needs of infection containment and addressing systemic gaps in infrastructure and surveillance [92]. The absence of a vaccine or specific antiviral treatment amplifies the importance of early diagnosis and strict infection control measures, including isolation and contact tracing. However, these strategies are resource-intensive and often constrained by socioeconomic factors, emphasizing the need for equitable investment in health systems. The World Health Organization’s classification of NiV as a priority pathogen underscores its potential to cause severe outbreaks with global ramifications. Despite this recognition, global efforts to combat NiV remain limited compared to other pathogens like Ebola or influenza. The lack of market incentives for vaccine development and antiviral research highlights a recurring challenge in addressing diseases with sporadic outbreaks but high lethality. Furthermore, the COVID-19 pandemic has demonstrated how interconnected health systems are vulnerable to novel pathogens, reinforcing the necessity of integrating Nipah surveillance into broader pandemic preparedness frameworks. While significant progress has been made in understanding NiV’s transmission dynamics and ecological drivers, critical gaps remain. For instance, improved surveillance of bat populations and ecological factors could enhance early warning systems. Similarly, research into potential antivirals and monoclonal antibodies holds promise but requires sustained funding and global collaboration. Innovative approaches, such as the development of pan-Henipavirus vaccines, could provide a broader protective strategy against related pathogens.

### A Call for Action

The Nipah virus serves as a stark reminder of the unpredictable nature of emerging infectious diseases and the importance of proactive measures to mitigate their impact. Strengthened global surveillance, coupled with investments in research and development, must become cornerstones of pandemic preparedness. Furthermore, fostering collaborations between governments, research institutions, and international organizations is vital to ensure a coordinated response to future outbreaks. As the global community continues to face an increasing number of zoonotic threats, the lessons from the Nipah virus emphasize the urgency of adopting a “One Health” approach, addressing the interconnectedness of human, animal, and environmental health. This integrated strategy is not only essential for combating Nipah but also for reducing the broader threat posed by emerging infectious diseases in the 21st century [94].

## 6. Limitations and Future Directions

While this review provides a comprehensive overview of the epidemiology, ecological factors, and public health responses to NiV, there remain critical gaps that require further investigation. A One Health approach—integrating human, animal, and environmental health—will be essential for understanding the complex drivers of NiV transmission. This includes exploring the role of climate change in altering bat habitats, zoonotic spillover risks, and the involvement of reservoirs beyond *Pteropus* bats. Another key limitation is the potential bias in sampling, particularly the underrepresentation of genomic sequences from under-researched regions. Addressing these gaps through targeted surveillance efforts in neglected areas is essential for achieving a more complete understanding of NiV transmission dynamics. Real-time data generation through advanced genomic surveillance and environmental monitoring can help detect early warning signals of potential spillovers. Coupling this with machine learning models offers a promising strategy to predict outbreak hotspots by leveraging environmental, ecological, and genomic data. Such predictive tools can inform targeted interventions and strengthen pandemic preparedness. By adopting a One Health framework, addressing sampling biases, and harnessing real-time data, future research can bridge existing knowledge gaps and provide actionable insights for mitigating the impact of this re-emerging zoonotic threat.

## Figures and Tables

**Table 1:** The key clinical differences between Malaysian and Bangladeshi NiV strains [26,40,43,44,45].

Feature	Malaysian NiV	Bangladeshi NiV
Geographic Distribution	Malaysia, Singapore, Philippines	Bangladesh, India (primarily)
Incubation Period	4–18 days	1–14 days (typically shorter, with a median of 5–6 days)
Onset of Illness	Often gradual, with prodromal symptoms	More often abrupt onset
Respiratory Symptoms	Less frequent; may include cough, sore throat	More prominent; often includes cough, difficulty breathing, and atypical pneumonia
Gastrointestinal Symptoms	Vomiting and diarrhea may occur	Vomiting and diarrhea are less common
Neurological Symptoms	Encephalitis, seizures, myoclonus (more common), and other neurological signs	Encephalitis, seizures, and other neurological signs (myoclonus less frequent)
Predominant Clinical Presentation	Encephalitis	Respiratory illness and encephalitis
Disease Progression	May progress more slowly	Often rapid progression to severe disease
Long-Term Sequelae	Less common; may include relapsing encephalitis	More common; may include neurological complications, such as seizures and personality changes
Mortality Rate	~40%	~70–100% (considerably higher)

**Table 2:** Current treatment strategies for NiV infection.

Drugs		Type of Study	Population	Intervention	Results	Ref.
Remdesivir		In vivo studies in animal model	Eight African green monkeys (AGM) (four treated and four controls) infected with NiV-B	Intravenous remdesivir administration for 12 days, starting within 24 h post-infection (early administration)	Mild, self-resolving respiratory symptoms in two of four treated AGMs. All controls developed severe respiratory symptoms, with two requiring euthanasia on day seven.	[66]
		In vivo studies in animal model	18 African green monkeys (AGM) (12 treated with different doses and 6 controls) infected with NiV-B	Intravenous remdesivir administration for 12 days, starting within 72 h post-infection (late administration)	Incomplete protection against severe Nipah virus, with a survival rate of 67%, was observed in the high-dose group. Treatment did not fully prevent the development of clinical signs, and surviving animals exhibited brain lesions upon histological examination.	[67]
Favipiravir		In vitro and in vivo studies in animal model	18 Syrian hamsters (10 treated and 8 control)	Twice daily 300 mg/kg/d favipiravir p.o. every 12 h, or 300 mg/kg/d once daily s.c., initiated immediately after infection and continued twice daily until 13 days post-infection	In vitro, favipiravir inhibited Nipah and Hendra virus replication and transcription at micromolar concentrations. In vivo, fully protected animals were challenged with a lethal dose of Nipah virus. While in vitro and in vivo studies demonstrated efficacy against NiV-M in hamsters, effectiveness against NiV-B remains unproven.	[68]
Ribavirin		Empirical anti-NiV strategy	12 patients (6 treated and 6 controls) during a 2018 NiV outbreak in India	Ribavirin therapy consisted of an initial dose of 2 g upon admission, followed by 1 g every 6 h for 4 days, and then 500 mg every 6 h for 6 days, administered orally.	A mortality rate of 100% was observed in the control group (six patients) and a morality rate of 66.7% was observed in the ribavirin-treated group (six patients). The small sample size limits definitive conclusions, and ribavirin’s use for NiV encephalitis remains debated.	[42]
		Open-label trial	194 patients (140 treated and 54 controls) between 1998 and 1999, from an NiV outbreak that occurred in Malaysia.	Oral ribavirin dosing schedule: 2 g on day 1, 1.2 g three times daily (tds) on days 2–4, 1.2 g twice daily (bd) on days 5–6, and 0.6 g bd for a further 1–4 days; patients unable to tolerate oral administration received intravenous ribavirin: a 30 mg/kg loading dose, followed by 16 mg/kg every 6 h for 4 days, and then 8 mg/kg every 8 h for 3 days.	A total of 45 deaths (32%) were observed in the ribavirin group and 29 deaths (54%) were observed in the control group, representing a statistically significant 36% reduction in mortality (*p* = 0.011) associated with ribavirin treatment. No serious side effects were observed.	[69]
		Case report	A 21-year-old male presented with 12 days of fever, altered sensorium, and cerebellar signs. NiV RT-PCR positive in CSF, throat swab, and urine	Treatment with ribavirin and immunoglobulins	Following treatment, the patient recovered and was discharged after 51 days of hospitalization.	[70]
		Case report	Five cases managed in hospital, during an NiV outbreak that occurred in India (2018)	Ribavirin	All patients developed encephalitis with viral bronchopneumonia/ARDS, and progressed to cardiorespiratory arrest and death.	[71]
		Case report	Eight healthcare workers (HCWs) exposed to NiV	Ribavirin 1000 mg thrice daily for 14 days started within 72 h from exposure	None of the HCWs contracted NiV disease.	[72]
Chloroquine		In vivo studies in animal model	Eight ferrets (six treated and two controls)	25 mg/kg/day of chloroquine (three ferrets before viral challenge; three ferrets 10 h after viral challenge; two controls).	Although chloroquine was effective in preventing the spread of NiV infection in vitro, it did not prevent the spread of NiV infection in vivo when used either as a prophylactic or a postexposure therapeutic.	[30]
		In vitro studies	293T (human kidney epithelial) and Vero (African green monkey kidney) cells	A high-throughput screening assay for inhibitors of infection based on envelope glycoprotein pseudotypes.	The study showed chloroquine’s ability to inhibit Hendra and Nipah virus infections by targeting Cathepsin L, an enzyme involved in viral fusion glycoprotein and virion maturation.	[73]
Monoclonal Antibodies	m102.4 (anti-HeV-G)	Double-blind, placebo–control-led, dose-escalation phase 1 trial	77 healthy adults enrolled in five cohorts. Within each cohort, a designated pair of participants was randomly assigned to receive either m102.4 or a placebo.	Participants in Cohorts 1 through 4 received a single intravenous infusion of m102.4 at escalating doses of 1, 3, 10, and 20 mg/kg, respectively, and were monitored for 113 days. Cohort 5 participants received two 20 mg/kg infusions, 72 h apart, and were monitored for 123 days.	Both single and repeated doses of m102.4 in healthy adult were as follows: ○ Well-tolerated and safe (no deaths or severe adverse events resulting in study discontinuation occurred). ○ Exhibited linear pharmacokinetics. ○ Elicited no detectable immunogenic response.	[74]
		In vivo studies in animal model	11 African Green Monkeys	2.5 × 10^5^ PFU intratracheal + 2.5 × 10^5^ PFU intranasal	While the human monoclonal antibody m102.4 has demonstrated efficacy in rescuing African green monkeys from Nipah virus Malaysia infection, its therapeutic window proved to be significantly shorter in AGMs infected with Nipah virus Bangladesh.	[40]
			16 African Green Monkeys (NiV-M)	5 × 10⁵ PFU intratracheal	All treated African green monkeys survived, while untreated controls died between days 8 and 10. Even when treatment was delayed until day 5 post-infection, all treated AGMs recovered by day 16, despite showing some clinical signs.	[75]
			Eight ferrets (NiV-M)	5 × 10^3^ TCID₅₀ oronasal	All ferrets treated with m102.4 ten hours after a high-dose oral–nasal Nipah virus challenge survived, whereas all untreated controls succumbed to the infection.	[76]
	h5B3.1 (anti-NiV-F)	In vivo studies in animal model	11 ferrets (NiV-M or HeV)	5 × 10^3^ PFU intranasal	All subjects treated with h5B3.1 between one and several days post-infection with a high-dose oral–nasal virus challenge survived. All untreated controls died.	[77]
	1F5 and 12B2 (anti-NiV-F)	In vivo studies in animal model	1° step: hamster model 2° step: 13 African green monkeys (NiV-B)	4 × 10^4^ PFU intranasal	1° step: After comparing hu1F5 and hu12B2 in a hamster model, hu1F5 demonstrated superior protection and was chosen over hu12B2 for further comparison with m102.4 in African green monkeys, using a stringent Nipah virus challenge. 2° step: While only one of six African green monkeys treated with m102.4 survived to the study endpoint, all six treated with hu1F5 were protected. Furthermore, even a reduced 10 mg/kg dose of hu1F5 provided complete protection against the Nipah virus challenge.	[78]
	NiV41 and NiV41-6 (anti-NiV-RBP)	In vivo studies in animal model	1° step: 12 hamster (NiV-B) 2° step: 48 hamster (NiV-M)	1° step: 10^5^ TCID50 intraperitoneal 2° step: 1000 LD50 intraperitoneal	In vivo testing shows that both NiV41 and its mature form (41-6) protect hamsters from the lethal Nipah virus challenge. A 2.88 Å Cryo-EM structure of the tetrameric RBP–antibody complex reveals that 41-6 blocks the receptor binding interface.	[79]
	Combination of anti-HeV-RBP	In vivo studies in animal model	HENV-26 and HENV-32 vs. 13 Ferrets ----- HENV-103, HENV-117, HENV-58, HENV-98, and HENV-100 vs. 46 Hamsters	5 × 10^3^ PFU intranasal ----- 5 × 10^6^ PFU intranasal	HENV-26 and HENV-32 protected ferrets against lethal Nipah virus Bangladesh infection when administered 3 days after exposure. ----- Reduced morbidity and mortality, and achieved synergistic protection when used in combination.	[80] --- [81]
Combination Therapies	Poly(I)-poly(C(12)U)	In vivo studies in animal model	Twelve golden hamsters (six controls)	Poly(I)-poly(C(12)U), at 3 mg/kg of body weight daily from the day of infection to 10 days postinfection	Poly(I)-poly(C(12)U), which induces IFN-α and IFN-β production, has shown complete in vitro inhibition of viral replication and prevented mortality in 83% of infected animals.	[82]
	Ribavirin plus Chloroquine	In vivo studies in animal model	15 golden hamsters	Three groups, each of five animals: (i) ribavirin, (ii) chloroquine, (iii) a combination therapy of ribavirin and chloroquine	Chloroquine provided no protection to hamsters, whether administered alone or in combination with Ribavirin. Ribavirin alone delayed death in NiV-infected hamsters by approximately 5 days.	[83]

**Table 3:** mRNA-based vaccine technologies for NiV: an overview [65].

Aspect	Details
Vaccine Platform	Messenger RNA (mRNA) technology, encoding viral glycoproteins (e.g., NiV G or F proteins) to elicit protective immune responses.
Mechanism of Action	mRNA instructs host cells to produce NiV glycoproteins, triggering both humoral (antibody-mediated) and cellular (T-cell) immune responses.
Target Antigens	Primarily NiV glycoproteins: G (attachment glycoprotein) and F (fusion glycoprotein). These antigens are crucial for viral entry into host cells.
Advantages	- Rapid development: mRNA vaccines can be designed and produced quickly in response to outbreaks. - Scalable production: mRNA vaccines are easier to manufacture at scale. - Safety: non-infectious, as they do not use live viruses. - Strong immune response: induces both neutralizing antibodies and T-cell immunity.
Challenges	- Stability: mRNA is sensitive to degradation and requires ultra-cold storage (70 °C to −20 °C). - Distribution infrastructure: limited storage and distribution capacity in low-resource settings. - Immune response durability: long-term protection data are still limited.
Immune Response Profile	- Strong neutralizing antibody response. - Robust T-cell-mediated immunity, particularly CD4+ and CD8+ T cells.
Global Collaboration	- CEPI (Coalition for Epidemic Preparedness Innovations) - WHO R&D Blueprint - Partnerships with academic institutions and biotech firms.
Regulatory Pathway	Fast-tracked under WHO Blueprint for Priority Pathogens, supported by CEPI (Coalition for Epidemic Preparedness Innovations).
Current Research Gaps	- Long-term immunity duration. - Cross-protection against emerging NiV strains. - Optimization of delivery Systems (E.G., Lipid nanoparticles).
Future Prospects	Integration of self-amplifying mRNA (saRNA) for enhanced immunogenicity and lower dosage requirements. Development of multivalent vaccines targeting multiple henipaviruses (e.g., NiV and HeV).
